# Wnt signaling directs human pluripotent stem cells into vascularized cardiac organoids with chamber-like structures

**DOI:** 10.3389/fbioe.2022.1059243

**Published:** 2022-11-18

**Authors:** Po-Yu Liang, Yun Chang, Gyuhyung Jin, Xiaojun Lian, Xiaoping Bao

**Affiliations:** ^1^ Davidson School of Chemical Engineering, Purdue University, West Lafayette, IN, United States; ^2^ Purdue University Center for Cancer Research, West Lafayette, IN, United States; ^3^ Department of Biomedical Engineering, The Huck Institutes of the Life Sciences, Department of Biology, The Pennsylvania State University, University Park, PA, United States

**Keywords:** human pluripotent stem cells, cardiomyocyte, cardiac endothelial cells, heart organoid, disease modeling

## Abstract

Heart diseases are leading cause of death around the world. Given their unique capacity to self-renew and differentiate into all types of somatic cells, human pluripotent stem cells (hPSCs) hold great promise for heart disease modeling and cardiotoxic drug screening. hPSC-derived cardiac organoids are emerging biomimetic models for studying heart development and cardiovascular diseases, but it remains challenging to make mature organoids with a native-like structure *in vitro*. In this study, temporal modulation of Wnt signaling pathway co-differentiated hPSCs into beating cardiomyocytes and cardiac endothelial-like cells in 3D organoids, resulting in cardiac endothelial-bounded chamber formation. These chambered cardiac organoids exhibited more mature membrane potential compared to cardiac organoids composed of only cardiomyocytes. Furthermore, a better response to toxic drugs was observed in chamber-contained cardiac organoids. In summary, spatiotemporal signaling pathway modulation may lead to more mature cardiac organoids for studying cardiovascular development and diseases.

## Introduction

Cardiovascular disorders, including congenital heart defects and cardiovascular disease, remain as a global health issue due to their high mortality worldwide (Balakumar and Kaur, 2009; Kadooka et al., 2021; Zhao et al., 2021). Developing new therapies is also challenging, since few drug candidates succeed to pass pre- or clinical trials and many of them are removed from the market due to severe cardiac toxicity (Balakumar and Kaur, 2009; Matoba and Egashira, 2011). To quickly screen effective and safe therapies, more authentic models of human heart are needed to be developed for pre-clinical research (Balakumar and Kaur, 2009; Matoba and Egashira, 2011; Jiang et al., 2017). For the past decades, animal model-based cardiac research has provided the basis for understanding heart development and diseases (Camacho et al., 2016). Due to the natural species difference, novel *in vitro* human cardiac models are urgently needed for a better understanding of tissue changes during development and diseases (Coales et al., 2016; Mathur et al., 2016; Veldhuizen et al., 2019). Human pluripotent stem cells (hPSCs) have been differentiated into cardiomyocytes (CMs) for heart disease modeling and cardiotoxic drug testing (Lian et al., 2013, 2015). However, commonly used two-dimensional (2D) culture models could not fully recapitulate the three-dimensional (3D) complexity of native human heart, in which the presence of various cardiovascular cell types in the defined geometry and cell-cell as well as cell-extracellular matrix (ECM) interactions play an essential role.

hPSCs are capable of differentiating and self-organizing into 3D tissue structures, termed organoids that resemble embryo-like tissues or organs (Drakhlis et al., 2021; Lewis-Israeli et al., 2021; Tan et al., 2021). While native-like organoid models have been widely implemented for various organs, the implementation of heart-like organoids remains challenging. Classic heart muscle engineering approaches that assemble pre-differentiated or primary cardiac cell types into 3D aggregates were commonly used to form cardiac microtissues that mimic adult-like heart tissue (Giacomelli et al., 2017). For example, Voges et al. (2017) and Mills et al. (2019) embedded hPSC-derived CMs in mixed collagen Ⅰ-Matrigel substrates to form cardiac organoids that reproduced some aspects of native human heart, including structured endothelial networks and epicardium. Vessel-like structures were observed in some areas of cardiac organoids, formed similarly by embedding hPSC aggregates in Matrigel (Drakhlis et al., 2021). Addition of exogenous VEGF in the cardioids has occasionally led to an intact endothelial layer that lines cardiac cavity (Hofbauer et al., 2021). Cardiac organoid studies that focus on native heart-like morphological aspects are still lacking. During embryo development, heart-forming progenitor cells extend across the midline and fuse into the heart tube, which then give rise to the myocardium and endocardium. After undergoing looping, the heart tube forms chambers (Tan and Lewandowski, 2020). To better recapitulate the complex native heart, there is a strong demand to bridge this technological and knowledge gap.

We have previously identified the temporal roles of canonical Wnt signaling during CM and epicardial cell differentiation from hPSCs (Bao et al., 2017b). Here, we first demonstrated reactivation of Wnt signaling at cardiac progenitor stage directed hPSCs into VE-cadherin (VE-cad)+ cardiac endothelial-like cells, which were incorporated into cardiac organoid differentiation and led to endothelial-bounded chamber formation. The resulting chambered cardiac organoids contained structured endothelial cells and CMs, and exhibited more mature membrane potential compared to cardiac organoids composed of only CMs. Furthermore, vascularized cardiac organoids were better in modeling cardiac response to toxic drugs. In summary, hPSC-derived vascularized cardiac organoids hold great potential for heart disease modeling and therapeutic drug screening.

## Materials and methods

### Maintenance of human pluripotent stem cells

H9 and H13 cell lines were purchased from WiCell and maintained on Matrigel- or iMatrix 511-coated plates in mTeSR plus medium at 37°C in humidified atmosphere with 5% CO_2_ according to our previously published method (Chang et al., 2022b).

### Hanging drop preparation for embryoid body formation

For embryoid body (EB) formation, 20 μl droplets were formed by pipetting on the inner side of a 96-well U-bottom culture plate lid. Droplets contained 100,000 hPSCs/ml and were composed of mTeSR Plus supplemented with 5 μM Y-27632 (Cayman Chemical) and iMatrx-511 (1:100). 100 μl PBS was added to each well before returning the lid to culture plate. After 24 h, EBs were transferred with a pipette to a new 96-well U-bottom culture plate and cultured in mTeSR Plus +5 μM Y-27632 for 1 day. The resulting EBs were then ready for further differentiation.

### Cardiovascular differentiation of hPSCs in 2D or 3D embryoid body cultures

For cardiac differentiation, hPSCs were seeded onto Matrigel-coated 6-well plate in mTeSR plus medium with 5 μM Y27632 at a cell density between 10,000 and 80,000 cell/cm^2^ after dissociating with 1 ml Versene solution. At day 0, hPSCs were treated with 6 μM CHIR99021 (Cayman Chemical) in RPMI for 24 h. At day 1 and day 2, the medium was switched to RPMI + B27 without insulin. At day 3 and day 4, 50% of medium was replaced with RPMI + B27 without insulin containing 4 μM Wnt-C59. At day 5, RPMI + B27 medium was used and changed every 3 days for CM differentiation. For cardiac organoid differentiation, EBs were treated with 9 μM CHIR99021 in RPMI medium for 24 h. At day 1 and day 2, the medium was switched to RPMI + B27-insulin medium. At day 3 and day 4, 50% of medium was replaced with RPMI + B27-insulin medium containing 4 μM Wnt-C59. At day 5, RPMI + B27 medium was used. At day 7, EBs were treated with EBM™ endothelial cell medium (Lonza) containing 50 μg VEGF (Peprotech) and 4.5 μM CHIR99021. At day 8 and day 9, medium change with EBM medium containing 50 μg VEGF. Between day 10 to day 15, EBs were treated with EBM medium containing 50 μg VEGF and 2.5 μM TGFβ inhibitor A83-001.

### Immunostaining and flow cytometry analysis

Immunostaining analysis was performed as described in an earlier publication (Chang et al., 2022a). Briefly, cells were fixed in PBS −/− containing 4% formaldehyde for 15 min in dark and incubated with appropriate antibodies in 5% nonfat dry-milk solution containing 0.4% Triton X-100. After gently washing and nuclei staining, cells were then imaged in a Leica DMi-8 microscope and processed in ImageJ. To collect differentiated cells for flow cytometry analysis, a 70 μm strainer was used to filter cells, followed by centrifugation and fixation in PBS −/− containing 1% formaldehyde for 20 min. The resulting cells were then permeabilized with ice-cold 90% methanol for at least 30 min and washed with PBS −/− solution containing 1% bovine serum albumin (BSA) and 0.1% Triton X-100. The cells were then stained with antibodies and analyzed in an Accuri C6 plus flow cytometry (Beckton Dickinson). Flow Jo software was used to process collected flow data.

### FluoVolt™ membrane potential kit analysis

Prepare labeling solution by adding FluoVolt™ dye (Invitrogen) into RPMI + B27 medium (1:50), and remove old medium from human heart organoid cultures. After washing heart organoids with PBS twice, organoids were treated with 100 μl of labeling solution under room temperature for 20 min. After removing dye solution and washing with PBS twice, organoids were kept in a 96-well U-bottom culture plate with 100 μl of PBS. The resulting cells were then live imaged in a Leica DMi-8 microscope.

### Hematoxylin and eosin staining of cardiac organoids

Differentiated cardiac organoids were fixed in 4% formaldehyde for 30 min, and embedded in paraffin. Slices with a thickness of 5 μm were incised for H&E staining, and the stained slice was imaged in a digital microscope.

### Cardiac organoids for drug treatment

The viability of cardiac organoids was assessed by the MTS assay. Differentiated cardiac organoids were incubated with 50 μg/ml of Temozolomide and subjected for MTS assay analysis at different time points. After Temozolomide treatment, old medium was aspirated and 100 μl of fresh culture medium containing 16.7% MTS stock solution was added into each well for 4 h at 37°C. The plates were then centrifuged at 2,000 rpm for 10 min. 80 μl supernatant in each cell culture well was pipetted into a well of 96-well microplate and measured at 490 nm for the absorbance of formazan through a SpectraMax iD3 microplate reader.

## Results and discussion

### Wnt signaling regulates cardiac endothelial cell specification

**FIGURE 1:**
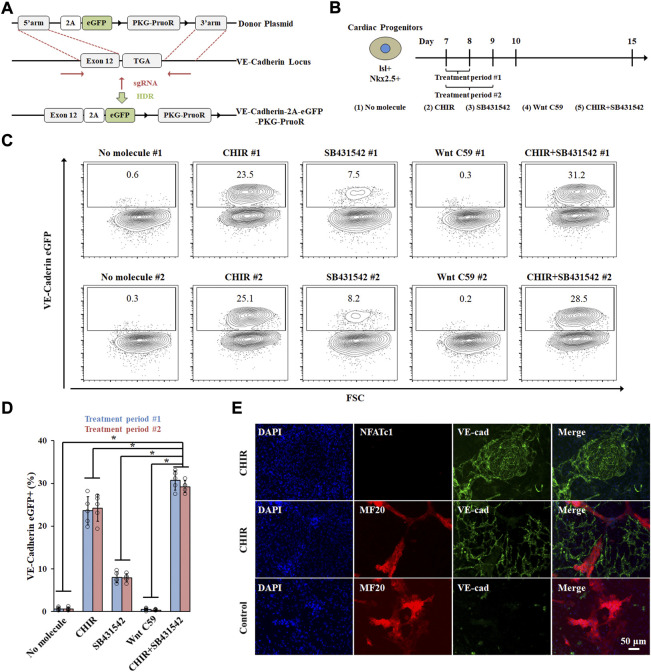
Cardiac endothelial cell generation by modulating Wnt signaling pathway. **(A)** Schematic illustration of knock-in strategy at the AAVS1 safe harbor locus. **(B)** Schematic of the protocol used to differentiate hPSCs towards cardiac endothelial cells. Conditions of 1–5 were applied to cardiovascular differentiation either from day 7–8 (period #1) or day 7–9 (period #2). VE-cad-GFP expression was analyzed by flow cytometry **(C)** and quantitated in **(D)**, n = 5, *p < 0.05. **(E)** Immunostaining images of cardiomyocytes and cardiac endothelial cells.

Cardiac endothelial cells arise from ISL1+NKX2.5 + progenitors during embryo development (Roden, 2013; Coales et al., 2016; Vermeulen et al., 2019) and we have previously identified ISL1+NKX2.5 + progenitor cells during cardiac differentiation from hPSCs (Bao et al., 2017c). To identify signaling pathways regulating cardiac endothelial cell specification, we used the H13 VE-cad-GFP reporter cell line that we previously made (Bao et al., 2017a) (Figure 1A) and expressed high levels of pluripotent markers (Supplementary Figures S1A–C). Day 6 cardiac progenitors derived from VE-cad-GFP reporter hPSCs were treated with different small molecules for 48 h (day 7 to day 9) (Figure 1B). CHIR99021 (CHIR), a GSK-3β inhibitor, significantly enhanced the expression of VE-Cad + cells (Figures 1C,D), which was blocked by a Wnt inhibitor Wnt-C59. Treatment with SB431542, a TGFβ signaling inhibitor, further promoted VE-Cad + cell generation (Figure 1D). The resulting cardiac endothelial cells did not express endocardium marker NFATc1 (Figure 1E), indicating their potential identity of cardiac microvascular endothelial cells. Notably, CHIR treatment from day 7–8 did not eliminate CM differentiation and led to co-differentiation of CM and cardiac endothelial cells, highlighting their application in multicellular cardiac organoid differentiation.

### Transcriptome analysis of hPSC-derived cardiac endothelial cells

**FIGURE 2:**
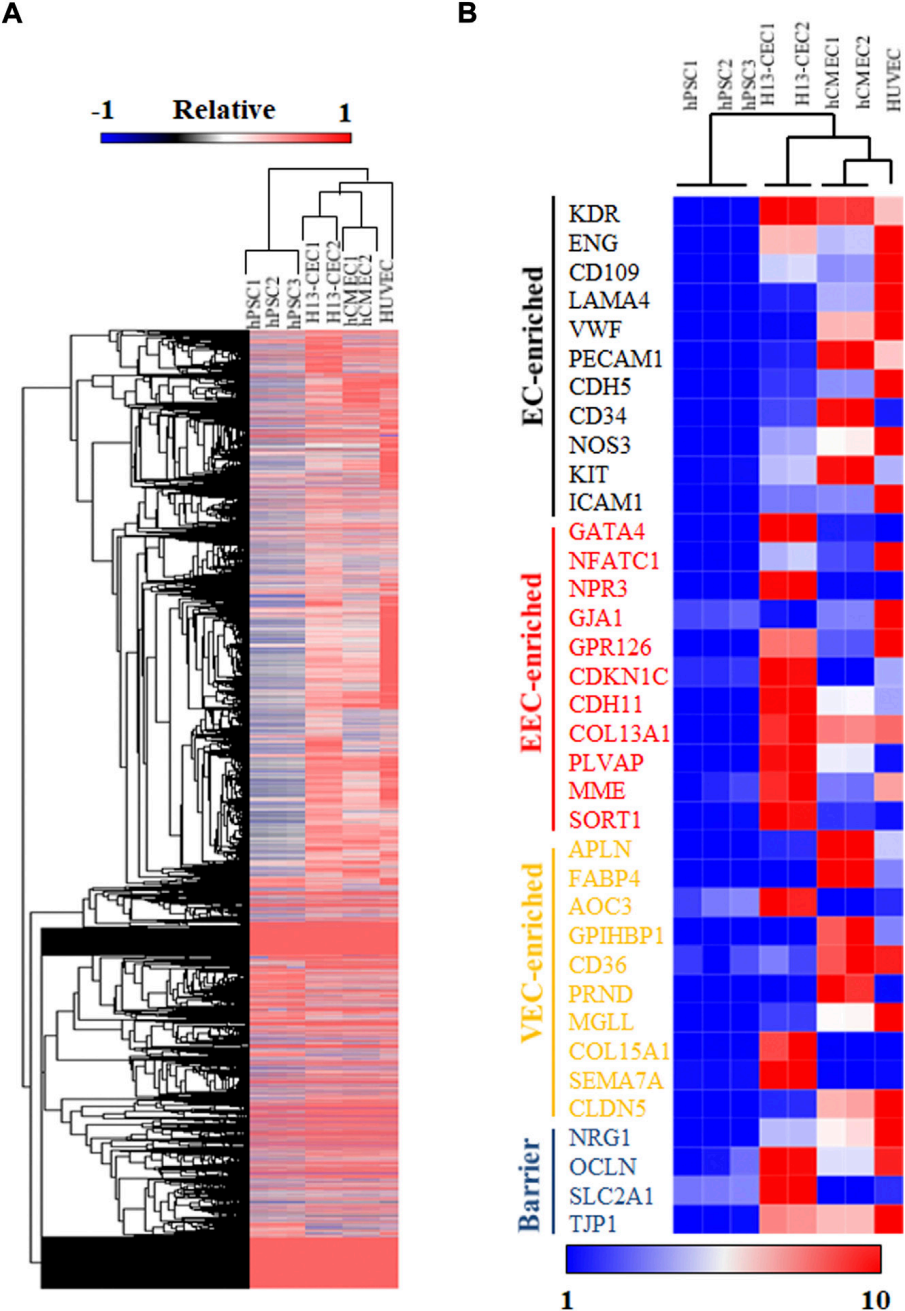
RNA sequencing (RNA-seq) analysis was performed on hPSC-differentiated cardiac endothelial cells (CECs). **(A)** Hierarchical clustering of RNA-seq expression data of hPSCs, H13-derived cardiac endothelial cells (CEC), primary human cardiac microvascular endothelial cells (hCMEC), and human umbilical vein endothelial cells (HUVEC). **(B)** Heatmap and hierarchical clustering analysis of selected endothelial cell markers.

To further confirm the identity of cardiac endothelial cells (CECs) derived from hPSCs, we sorted day 15 VE-cad + cells and performed bulk RNA sequencing (RNA-seq) analysis. Hierarchical clustering analysis of RNA-seq expression data demonstrated that day 15 CECs were closely clustered to primary human cardiac microvascular endothelial cells (hCMEC) (Figure 2A). Notably, examination of specific endocardial endothelial cell (EEC) genes revealed that hPSC-derived CECs displayed higher expression levels of EEC and barrier makers as compared to hCMEC and HUVEC (Figure 2B). Hierarchical clustering analysis revealed all endothelial cell populations were clustered together as compared to undifferentiated hPSCs, though hPSC-derived CECs were relatively far away from primary hCMEC and HUVEC, indicating the relative immaturity of hPSC-derived CECs. Consistent with this data, relatively lower expression levels of general endothelial and vascular endothelial cell (VEC) markers were also observed in hPSC-derived CECs. In summary, bulk RNA-seq analysis suggested the similarity between our hPSC-derived CECs and primary hCMEC in global transcriptome expression.

### Vascularized cardiac organoids derived from hPSCs

**FIGURE 3:**
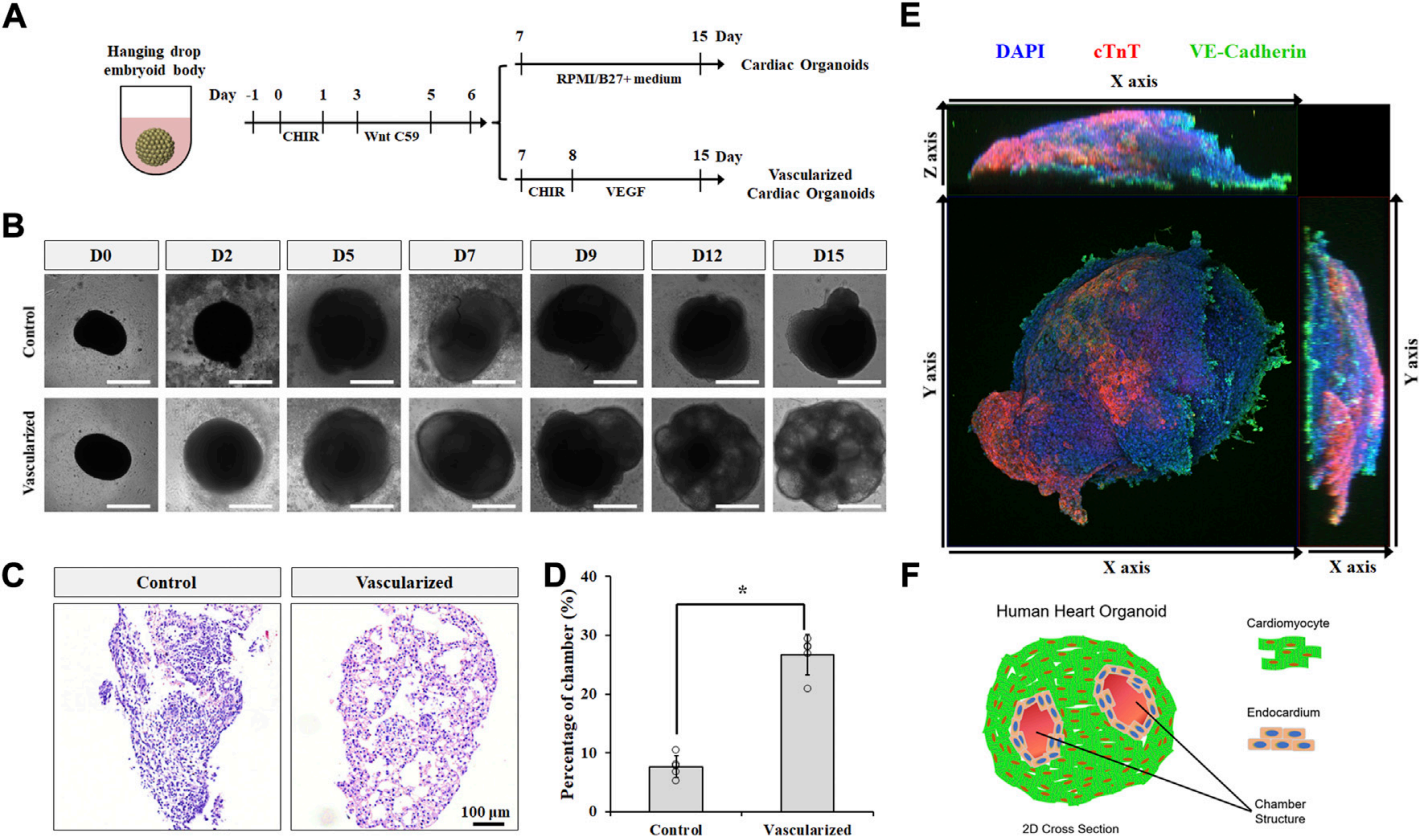
Vascularized cardiac organoids were derived from hPSCs. **(A)** Schematic of the protocol used to differentiate hPSCs towards vascularized cardiac organoids. **(B)** Brightfield images of hPSC-derived cardiac organoids at indicated days. **(C)** H&E staining showed sliced cardiac organoids. **(D)** Chamber area quantification in different cardiac organoids. n = 4. Confocal microscope image **(E)** and illustration **(F)** of vascularized cardiac organoids.

To better recapitulate multicellular heart development *in vivo*, we incorporated our 2D co-differentiation protocol into the classic hanging drop platform for embryoid body (EB) formation in 3D. hPSC EBs were cultured and expanded for 2 days prior to cardiac differentiation. Similar to 2D monolayer culture, GiWi method was used to induce cardiac progenitor generation in 3D EBs (Figure 3A). After day 6, EBs were treated with CHIR and vascular endothelial growth factor (VEGF) to form vascularized cardiac organoids *via* co-differentiation of CMs and CECs in 3D, or left untreated to form regular cardiac organoids composed of CMs only. VEGF is a commonly used growth factor for inducing vascularization in tissues (Shibuya, 2011; Poldervaart et al., 2014). While both kinds of organoids showed a significant increase in size throughout the differentiation protocol (Figure 3B and Supplementary Figure S2A), vascularized cardiac organoids displayed obvious chamber formation, which was also confirmed by H&E staining of sliced organoids (Figures 3C,D, Supplementary Figure S2B). Immunostaining analysis on sliced cardiac organoids confirmed the presence of structured endothelial cells and CMs in 3D (Figure 3E). Flow cytometry analysis showed about 2% CECs and 62% CMs, and 19% CECs and 48% CMs presented in control and vascularized organoids (Supplementary Figures S3A,B), respectively. Overall, the resulting vascularized cardiac organoids contained significantly more chambers, mimicking the anatomical structure of the developing embryonic heart (Figure 3F).

### hPSC-derived vascularized cardiac organoids better respond to cardiotoxic drugs

**FIGURE 4:**
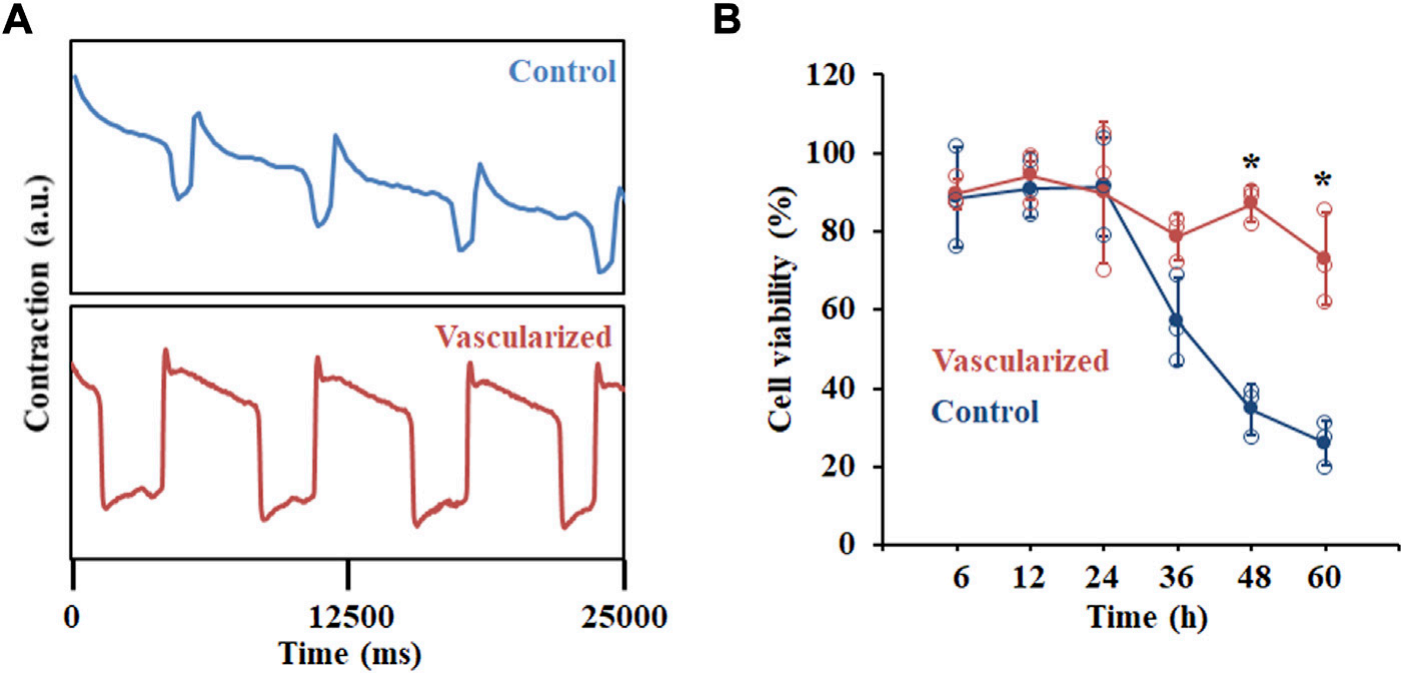
Functional analysis of hPSC-derived cardiac organoids. **(A)** Representative contraction amplitude plots of hPSC-derived cardiac organoids using a voltage dye. **(B)** Cell viability analysis of hPSC-derived cardiac organoids after Temozolomide treatment at indicated time points. n = 3.

A critical feature of *in vitro*-derived organoids is to recapitulate at least one specialized aspect of the native tissues or organs. In terms of cardiac tissue, the contractile activity of hPSC-derived cardiac organoids is an important function. We first analyzed the beating rates of our hPSC-derived cardiac organoids with a highly sensitive voltage dye (Figure 4A). Compared with CM organoids, vascularized cardiac organoids contain more cell types with human heart-like layered structures. In addition, CECs were reported to promote the trabeculation and maturation of CMs (Mikryukov et al., 2021). All these additional features of our vascularized cardiac organoids may lead to a better cardiac model for cardiotoxic drug testing. To test this hypothesis, we studied the possibility of modelling chemotherapy drug-induced cardiotoxicity with cardiac organoids derived from hPSCs by treating them with supernatant medium of chemical drug Temozolomide (Temo)-treated tumor cells. This drug was widely used for glioblastoma treatment, which though induced cardiotoxic and pro-fibrotic effects, causing or exacerbating heart failure. hPSC-derived cardiac organoids were treated with 0.2 and 2 μg/ml Temo, which broke the integral morphology of cardiac organoids (Supplementary Figure S4), possibly due to cell death induced by the drug (Figure 4B).

## Conlusion

In this study, we identified that Wnt signaling regulates hPSC differentiation into cardiac endothelial cells (CECs) *in vitro*, consistent with its role in native heart development *in vivo*. (Mukhopadhyay, 2021). Modulation of Wnt signaling also enabled the co-differentiation of CM and CEC differentiation in 2D and 3D cultures. Notably, cardiac organoids treated with the Wnt activator CHIR99021 displayed more chamber formation and more mature beating curve compared with cardiac organoids composed of only CMs. The resulting vascularized cardiac organoids also served as a better model for toxicity analysis of antitumor drugs. In conclusion, hPSC-derived vascularized cardiac organoids hold great potential for heart disease modeling and therapeutic drug screening.

## Data Availability

The datasets presented in this study can be found in online repositories. The names of the repository/repositories and accession number(s) can be found in the article/Supplementary Material.
