# Supplementary material for: Wnt signaling directs human pluripotent stem cells into vascularized cardiac organoids with chamber-like structures
Source: Front Bioeng Biotechnol. 2022 Nov 18;10:1059243. doi: 10.3389/fbioe.2022.1059243 (PMC9715615; doi:10.3389/fbioe.2022.1059243)
Supplement: Supplementary file 1 [file DataSheet1.pdf]

Supplementary Material

Wnt Signaling Directs Human Pluripotent Stem Cells into Vascularized Cardiac Organoids with Chamber-like Structures

Po-Yu Liang[1#], Yun Chang[1,2#], Gyuhyung Jin[1,2], Xiaojun Lian[3,*], Xiaoping Bao[1,2*]

[1]Dacidson School of Chemical Engineering, Purdue University, West Lafayette, IN, USA

[2]Purdue University Center for Cancer Research, West Lafayette, IN, USA

[3]Department of Biomedical Engineering, The Huck Institutes of the Life Sciences, Department of Biology, The Pennsylvania State University, University Park, PA,USA

*** Correspondence:**
Xiaojun Lian
lian@psu.edu

Xiaoping Bao
bao61@purdue.edu

[#]These authors contributed equally

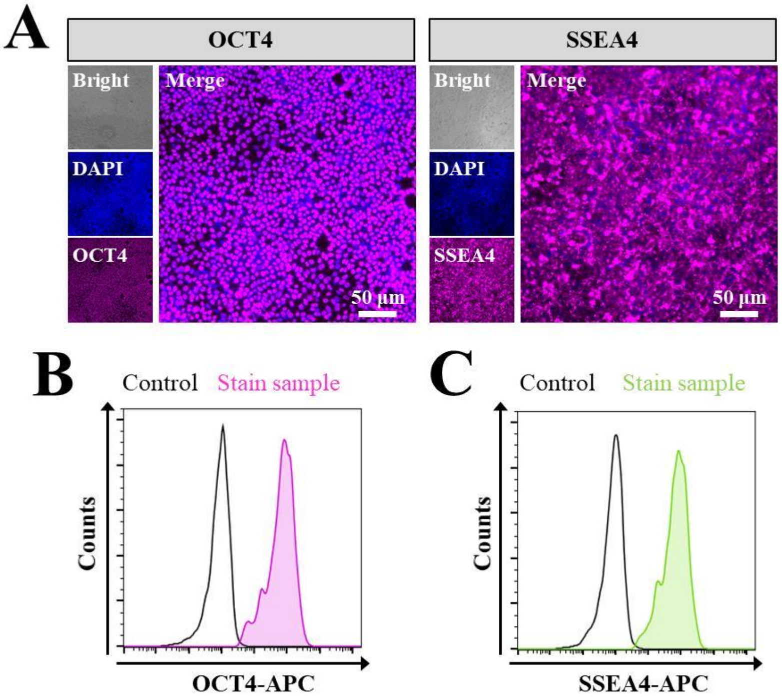

Supplementary Figure 1. Pluripotent stem cell marker characterization. (A) Fluorescence images of OCT4 and SSEA4 expression. Flow analysis of OCT4 (B) and SSEA4 (C) expression.

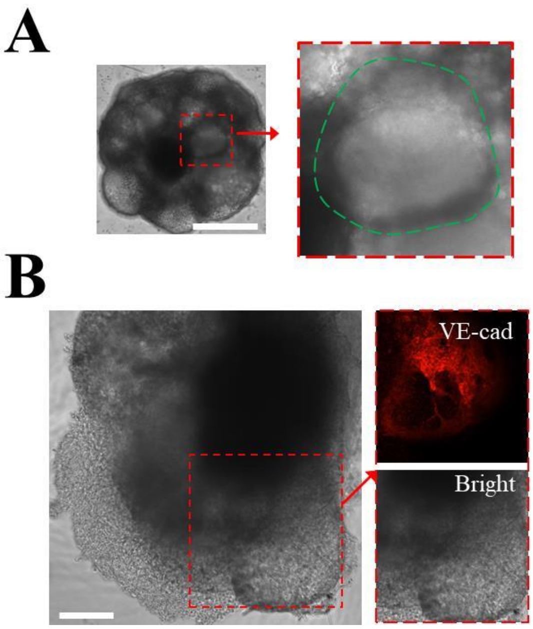

Supplementary Figure 2. Chamber characterization. (A) Brightfield images of cardiac organoids. (B) Representative fluorescence images of VE-cad expression inside cardiac organoids.

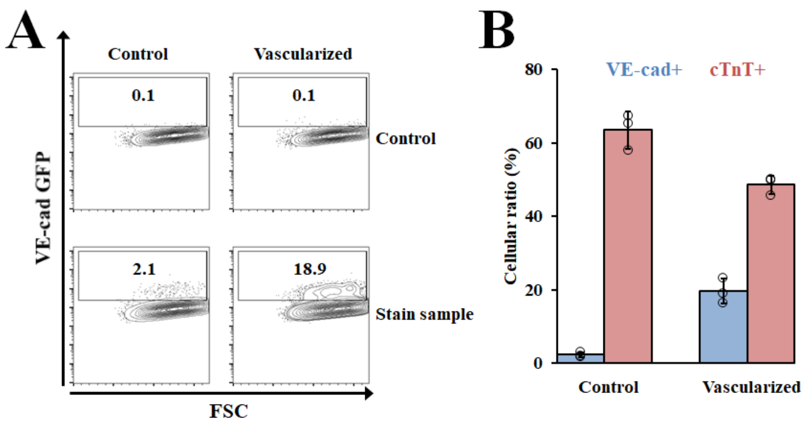

Supplementary Figure 3. Flow cytometry analysis of cardiac endothelial cells (CECs) and cardiomyocytes (CMs) in hPSC-derived organoids. (A) Representative flow cytometry analysis of VE-cad-GFP expression within the indicated cardiac organoids was shown. (B) The percentages of CECs and CMs within indicated cardiac organoids were quantified and shown.

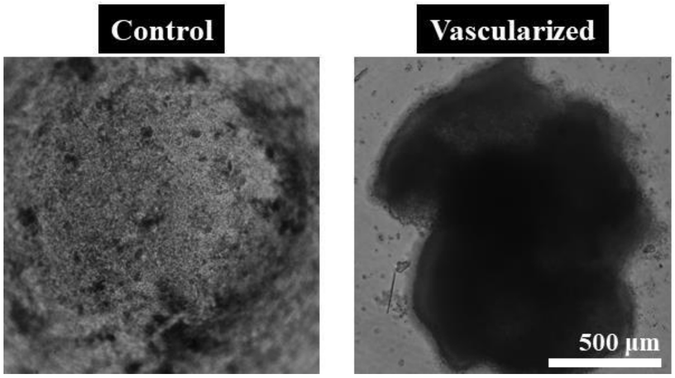

Supplementary Figure 4. Brightfield images of different cardiac organoids after the treatment with Temozolomide.
